# Philadelphia chromosome-negative B-cell acute lymphoblastic leukaemia with kinase fusions in Taiwan

**DOI:** 10.1038/s41598-021-85213-6

**Published:** 2021-03-11

**Authors:** Yin-Chen Hsu, Chih-Hsiang Yu, Yan-Ming Chen, Kathryn G. Roberts, Yu-Ling Ni, Kai-Hsin Lin, Shiann-Tarng Jou, Meng-Yao Lu, Shu-Huey Chen, Kang-Hsi Wu, Hsiu-Hao Chang, Dong-Tsamn Lin, Shu-Wha Lin, Ze-Shiang Lin, Wei-Tzu Chiu, Chia-Ching Chang, Bing-Ching Ho, Charles G. Mullighan, Sung-Liang Yu, Yung-Li Yang

**Affiliations:** 1 Department of Clinical Laboratory Sciences and Medical Biotechnology, College of Medicine, National Taiwan University, No. 1, Changde Street, Zhongzheng District 10048, Taipei, Taiwan; 2 Department of Pathology, St. Jude Children’s Research Hospital, 262 Danny Thomas Place, Memphis, TN 38105-3678 USA; 3 Department of Laboratory Medicine, National Taiwan University Hospital, 100, No 7, Chung Shan South Road, Taipei, Taiwan; 4 Department of Pediatrics, National Taiwan University Hospital, Taipei, Taiwan; 5 Department of Pediatrics, College of Medicine, National Taiwan University, Taipei, Taiwan; 6 Department of Pediatrics, Taipei Medical University–Shuang Ho Hospital, Taipei, Taiwan; 7 Department of Pediatrics, Chung Shan Medical University Hospital and School of Medicine, Chung Shan Medical University, Taichung, Taiwan; 8 Institute of Medical Device and Imaging, College of Medicine, National Taiwan University, Taipei, Taiwan; 9 Centers of Genomic and Precision Medicine, National Taiwan University, Taipei, Taiwan; 10 Graduate Institute of Pathology, College of Medicine, National Taiwan University, Taipei, Taiwan; 11 Department of Laboratory Medicine, College of Medicine, National Taiwan University, Taipei, Taiwan

**Keywords:** Haematological cancer, Paediatric cancer

## Abstract

Philadelphia chromosome-like (Ph-like) acute lymphoblastic leukaemia (ALL), a high-risk subtype characterised by genomic alterations that activate cytokine receptor and kinase signalling, is associated with inferior outcomes in most childhood ALL clinical trials. Half of the patients with Ph-like ALL have kinase rearrangements or fusions. We examined the frequency and spectrum of these fusions using a retrospective cohort of 212 newly diagnosed patients with childhood B-cell ALL. Samples without known chromosomal alterations were subject to multiplex reverse transcription polymerase chain reaction to identify known Ph-like kinase fusions. Immunoglobulin heavy chain locus (IGH) capture and kinase capture were applied to samples without known kinase fusions. We detected known kinase fusions in five of 212 patients, comprising *EBF1-PDGFRB, ETV6-ABL1, ZC3HAV1-ABL2, EPOR-IGH*, and *CNTRL-ABL1*. Two patients with *P2RY8-CRLF2* were identified. Patients with non-Ph kinase fusions had inferior 5-year event-free survival and overall survival compared with patients with other common genetic alterations. The prevalence of non-Ph kinase fusions in our Taiwanese cohort was lower than that reported in Caucasian populations. Future clinical trials with tyrosine kinase inhibitors may be indicated in Taiwan because of the inferior outcomes for B-cell ALL with kinase fusions.

## Introduction

Multiple genetic subtypes have prognostic implications and clinical significance in acute lymphoblastic leukaemia (ALL). These ALL-associated genetic alterations include aneuploidy, copy number variations, sequence mutations, and chromosomal translocations that result in aberrant expressions of oncogenes or the construction of chimeric fusion genes^1^. Advances in cancer genomics and large-scale integrated transcriptome analyses have markedly expanded our knowledge of the genetic landscape of ALL via the classification of new subtypes in recent years^2–16^. One high-risk subtype is Philadelphia chromosome-like (Ph-like) ALL, highlighted by a gene expression profile similar to that of Ph-ALL, but without the *BCR-ABL1* fusion^17–19^. Approximately 15% of children and 20–25% of adolescent and young adult B-ALLs are Ph-like, which is associated with inferior event-free survival (EFS) and overall survival (OS) compared with that of non-Ph-like ALL in most studies^3,20–24^. Other studies also demonstrated that adult ALL patients with Ph-like were susceptible to having residual disease persistence and poor outcomes^25,26^. Most patients with Ph-like ALL have somatic gene alterations that activate kinase and cytokine receptor signalling^3,4,27^.

Roberts et al. profiled the genomic alterations of Ph-like ALL in children, adolescents, and young adults using transcriptome analyses^3,4,21,22^. Approximately half patients with Ph-like disease overexpress cytokine receptor-like factor 2 (*CRLF2*). The overexpression of *CRLF2* originates from a rearrangement that fuses the immunoglobulin heavy chain locus (IGH) or the *P2RY8* gene promoter with *CRLF2*^28–32^. The remaining patients with Ph-like ALL have a variety of kinase alterations, including fusions involving ABL-class genes (*ABL1, ABL2, CSF1R, PDGFRB*, and *PDGFRA*) that are sensitive to ABL1 tyrosine kinase inhibitors (TKIs), rearrangements that create JAK2 fusion proteins or truncating rearrangements of the erythropoietin receptor (EPOR) that are sensitive to ruxolitinib in vitro^3,4,21,27,33^. *NTRK3*, *PTK2B* and other kinases are also involved in several patients^3,20–22^.

Unlike Ph-positive and other subtypes of B-cell ALL that are characterised by recurrent, defined chromosomal rearrangement break points, Ph-like ALL is defined by its gene expression profile (GEP), and has a heterogeneous collection of rearrangements or genetic alterations, making the diagnosis and identification of these rearrangements a medical challenge^17–19^. Reshmi et al. used low-density array (LDA) to screen Ph-like ALL, followed by multiplex reverse transcription (RT) polymerase chain reaction (PCR) to identify known Ph-like kinase fusions. They also used kinome kits with next-generation sequencing (NGS) or RNA-seq on Ph-like ALL samples without known kinase fusions and demonstrated the feasibility of their strategy in clinical practice^21^. The identification of these kinase fusions is vital for the possible addition of TKI to chemotherapy in clinical trials^34^.

GEP may be required to diagnose Ph-like ALL, followed by FISH, target sequencing, or RNA-seq to identify the precise kinase fusion genes. Comprehensive genome analysis previously showed that most genetic subtypes of B-ALL are mutually exclusive^16^. Cytogenetics and RT-PCRs can exclude the major known subtypes of B-ALL, such as *ETV6-RUNX1*, *TCF3-PBX1*, hyperdiploidy, *BCR-ABL1* and *KMT2A* fusions. Therefore, we modified the strategy proposed by Reshmi et al. to identify the kinase fusions in childhood B-ALL in this retrospective study^21^.

## Materials and methods

### Patients and protocols

Between January 2002 and December 2016, 354 children with B-ALL were included in this study, which was performed at the National Taiwan University Hospital. Adequate diagnostic bone marrow or peripheral blood samples were obtained from 212 children with B-ALL, whereas 142 patients were excluded from this study because of unavailability of diagnostic samples. The diagnosis of ALL was based on the morphologic examination of the bone marrow aspirates and immuno-phenotype analysis of leukaemic cells by flow cytometry. Conventional cytogenetic analyses were performed as part of the routine work-up. The patients were enrolled in the TPOG ALL 2002 or TPOG ALL 2013 protocols. These protocols also included chemotherapy for infantile ALL. TPOG ALL 2013 modified the TPOG ALL 2002 protocol by incorporating minimal residual disease measurements to stratify treatment. The Institutional Review Board of National Taiwan University Hospital approved the study, and all of the participants or their guardians provided written informed consent in accordance with the Declaration of Helsinki. Details of the protocols and risk group assignment have been published elsewhere^35–37^.

### Multiplex ligation-dependent probe amplification analysis

We analysed genomic DNA after multiplex ligation-dependent probe amplification (MLPA) using the SALSA MLPA kit (MRC-Holland, Amsterdam, Netherlands), according to the manufacturer’s instructions. The PCR fragments were separated by capillary electrophoresis using Life Technologies 3500 Genetic Analyzer (Thermo Fisher Scientific, Waltham, MA, USA). We analysed the MLPA data using Coffalyser.Net v.140721.1958 (MRC-Holland, Amsterdam, Netherlands). Probe ratios between 0.75 and 1.3 were considered within the normal range. A probe ratio of < 0.75 or > 1.3 indicated deletion or gain, respectively. A probe ratio of < 0.25 or > 1.8 indicated biallelic deletion or amplification. We used the SALSA MLPA P335 ALL-IKZF1 probemix to detect alterations of *EBF1*, *IKZF1*, *CDKN2A*, *CDKN2B*, *PAX5*, *ETV6*, *RB1* and *BTG1*. We used SALSA MLPA P327 iAMP21-ERG probemix to detect alterations in *ERG* and the intrachromosomal amplification of chromosome 21. We used SALSA MLPA P329 CRLF2-CSF2RA-IL3RA probemix to detect *P2RY8-CRLF2* (PAR1 deletion)^38^.

### RT-PCR for non-Ph kinase fusions

**Figure 1:**
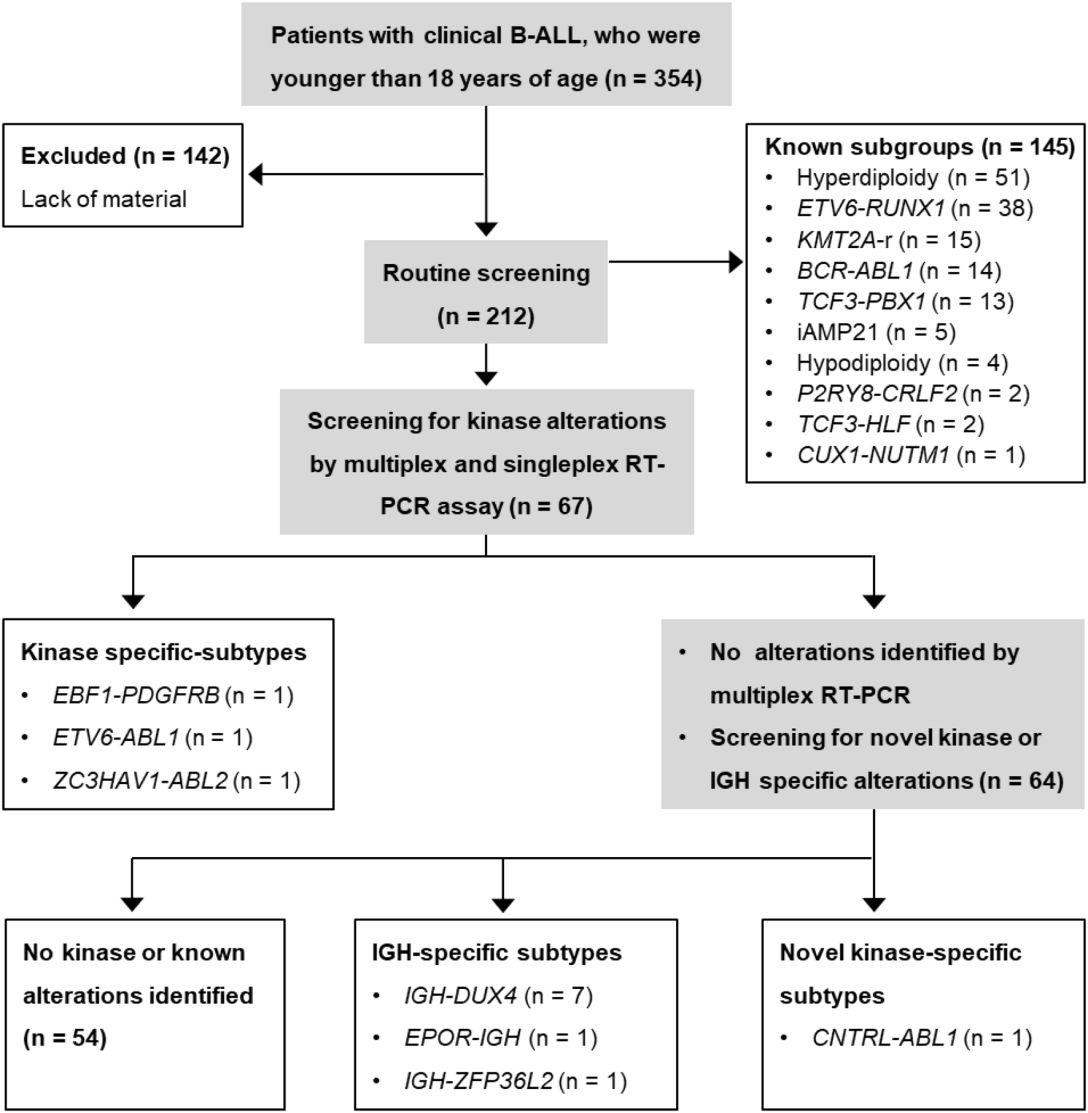
Testing algorithm for identifying kinase alterations in this cohort.

Most major cytogenetic alterations in childhood B-ALL are mutually exclusive^16^. The study design for this project is shown in Fig. 1. We screened using RT-PCR to identify samples with *ETV6-RUNX1*, *TCF3-PBX1*, *BCR-ABL1* and *P2RY8-CRLF2*. The primer sequences for these fusions are listed in the Supplementary Table S1. The patient samples without known cytogenetic alterations were subject to multiplex and singleplex RT-PCR assays for previously identified kinase fusions. In brief, we prepared complementary DNA (cDNA) from total RNA (1 µg) using High-Capacity cDNA Reverse Transcription Kit (Life Technologies, Waltham, MA, USA). We then used the cDNA (0.5 µL; equivalent to 25 ng of starting RNA) for RT-PCR amplifications to detect kinase fusions occurring in *ABL1, ABL2, CSF1R, JAK2, NTRK3, TYK2* and *PDGFRB*. The primer sequences were published by Reshmi et al.^21^.

### Detection of fusion transcripts using RNA kinome capture sequencing

Patient samples with no kinase gene fusions identified by multiplex or singleplex PCR were subject to RNA kinome capture sequencing of 612 genes (571 kinases) using Agilent SureSelect RNA Kinome Capture Kit (Agilent Technologies, Santa Clara, CA, USA). The RNA library was prepared per the manufacturer’s instructions and sequenced using Illumina NextSeq 500. To detect kinase fusions, samples were analysed using the Rapid RNA-Seq tool from the St. Jude Cloud (https://www.biorxiv.org/content/10.1101/2020.08.24.264614v1)^39^. All identified fusions were confirmed via bidirectional Sanger sequencing.

### IGH capture sequencing

IGH capture sequencing was performed using a SeqCap EZ product from Roche/NimbleDesign (Roche Sequencing Solutions, Pleasanton, CA, USA). The custom region (~ 3 Mb) was designed to capture 17 genes including *IGH* and 16 additional *IGH*-related partner genes. The genes are listed in Supplementary Table S2. We prepared the DNA library as per the manufacturer’s instructions and sequenced using Illumina NextSeq 500. To detect IGH translocations, we used the Fusion and Chromosomal Translocation Enumeration and Recovery Algorithm for the detection of genomic fusions in paired-end targeted sequencing data^40^. The primer sequences for the validated fusions are listed in the Supplementary Table S1.

### Statistical analysis

Five-year EFS and OS were calculated using the Kaplan–Meier method, and the log-rank test was used to compare differences between survival curves^41^. The OS was measured from the protocol commencement date until the date of death regardless of cause, excluding patients who were alive at the last follow-up. EFS was defined as achieving complete remission, and was measured from the date of attaining complete remission until the date of relapse. Patients with no reports of relapse by the end of the follow-up were censored on the date of last follow-up. Fisher’s exact test was used for the analysis of contingency tables. Comparisons of the distribution of continuous variables in different groups including age at the time of onset and white blood cell counts were performed using the Mann–Whitney test. An effect was considered significant if the *P* value was < 0.05. The statistical software SAS 9.4 (SAS Institute, Cary, NC, USA) was used for all data analyses.

### Ethics declaration

This study was conducted in accordance with the Declaration of Helsinki guidelines. Written informed consent was obtained from all study participants or their guardians.

## Results

### Detecting kinase fusion genes using multiplex RT-PCR

**Figure 2:**
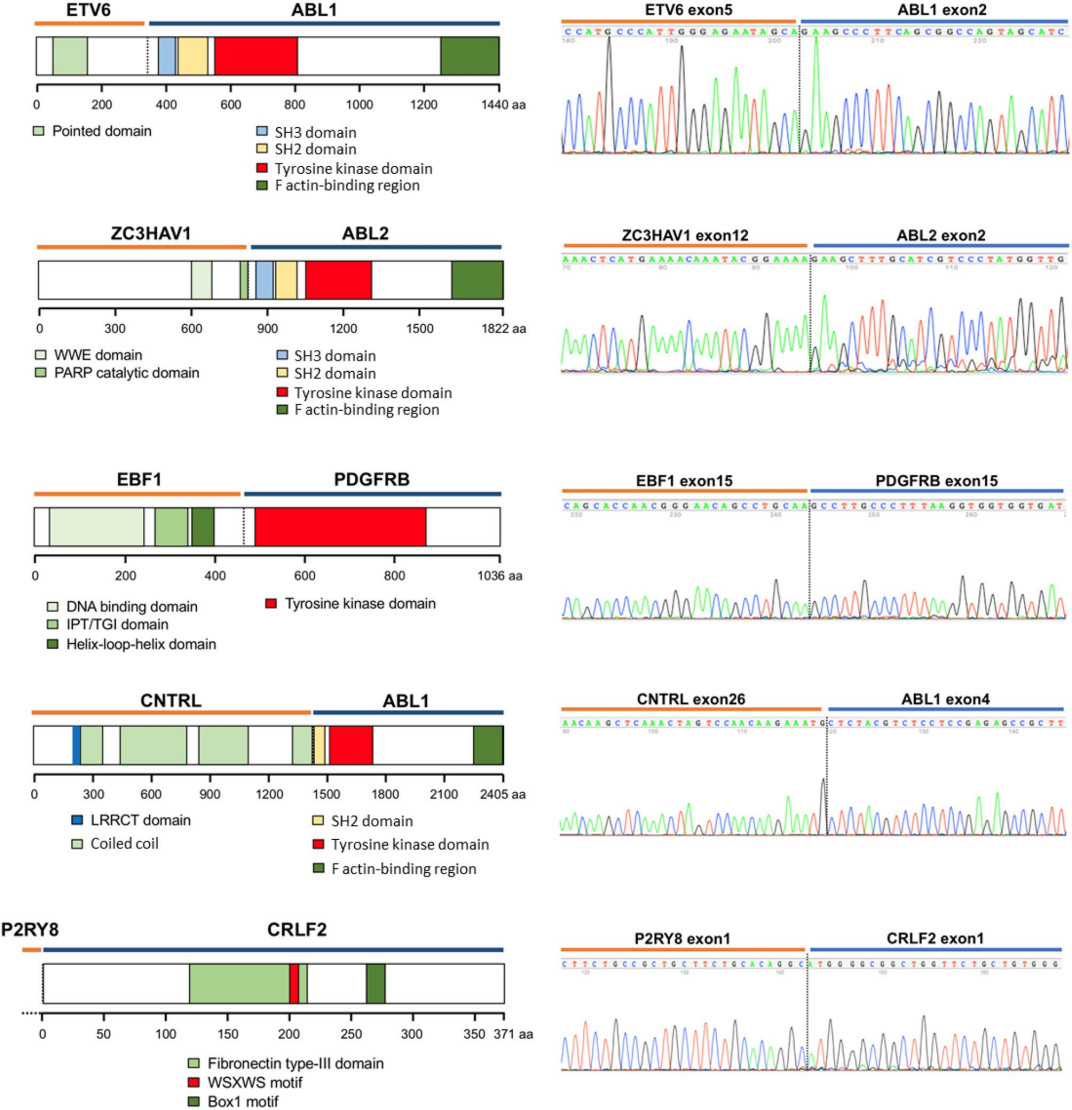
Validated Sanger sequences and protein domains of detected kinase fusions.

A total of 142 patients were excluded from the study because of the lack of diagnostic samples. The clinical characteristics of studied and non-studied cohorts are summarized in Supplementary Table S3. The white blood cell counts were lower in the studied subjects than in the non-studied cohort. The clinical features of the entire studied cohort are summarized in Table 1. Of 212 patients, 145 were not subject to further multiplex RT-PCR or next-generation sequencing assays as they carried known ALL subtype genetic alterations, including hyperdiploidy, hypodiploidy, intrachromosomal amplification of chromosome 21, *KMT2A* rearrangement, *ETV6-RUNX1*, *BCR-ABL1*, *TCF3-PBX1*, *TCF3-HLF* and *P2RY8-CRLF2*. A schematic diagram illustrating this study is shown in Fig. 1. The remaining 67 patients were analysed for additional kinase alterations, initially using RT-PCR for known fusions and, if negative, by kinome capture followed by NGS. We discovered 3 in-frame fusion candidates—*EBF1-PDGFRB*, *ETV6-ABL1* and *ZC3HAV1-ABL2*—which we validated by Sanger sequencing. The validated Sanger sequences are listed in Fig. 2. Sixty-four samples tested negative by PCR analysis were subject to kinome analysis. We detected a novel *CNTRL-ABL1* fusion by kinome analysis, which we validated by Sanger sequencing (Fig. 2).

**Table 1:** Clinical characteristics of the cohort categorized by *BCR-ABL1*, non-Ph kinase fusion, and *DUX4* rearrangement status.

Characteristics	Total		Others		*BCR-ABL1*			Non-Ph kinase fusion			*DUX4*-r		
	n = 212	%	n = 184	%	n = 14	%	P-value^a^	n = 7	%	P-value^a^	N = 7	%	P-value^a^
**Age (years)**							< 0.0001			0.07			0.005
< 1	11	5.2	11	6.0	0	0		0	0		0	0	
1–9	144	67.9	135	73.4	3	21.4		3	42.9		3	42.9	
> 10	57	26.9	38	20.6	11	78.6		4	57.1		4	57.1	
Median (range)	5.3 (0.01–18)		4.6 (0.01–17.5)		14.7 (4.8–18.0)			15.0 (2.7–17.6)			10.4 (5.9–17.4)		
**Gender**							0.27			0.02			0.70
Male	121	57.1	101	54.9	10	71.4		7	100		3	42.9	
Female	91	42.9	83	45.1	4	28.6		0	0		4	57.1	
**WBC (× 10**^**9**^**/L)**							0.0002			0.014			0.21
< 50	151	71.2	139	75.6	3	21.4		3	42.8		6	85.7	
50–100	27	12.7	19	10.3	5	35.7		2	28.6		1	14.3	
> 100	34	16.1	26	14.1	6	42.9		2	28.6		0	0	
Median (range)	18.6 (0.6–1173)		16.3 (0.6–1173)		94.3 (4.0–670)			67.1 (7.3–432)			11.1 (1.7–54.9)		
**Gene deletion**													
*IKZF1* deletion^b^	36	21.6	18	12.5	9	90	< 0.0001	4	66.7	0.004	5	71.4	0.0009
*EBF1* deletion^b^	11	6.6	9	6.3	1	10	0.99	1	16.7	0.34	0	0	1.00
Data not available	45		40		4		–	1		–	0		–

*WBC* white blood cells, *-r* rearrangements.

^a^P-value was calculated using Fisher’s exact test or Mann–Whitney U test for age and WBC between each subtype and “Others.”

^b^The prevalence was calculated amongst patients with data available (n = 167).

### IGH fusions identified using IGH capture-based NGS

The procedures described above were not able to identify *EPOR*-*IGH* and *IGH-CRLF2* rearrangements. Therefore, we used IGH capture method, followed by NGS. A patient with known *IGH* rearrangements, such as those found in Burkitt’s leukaemia, served as the positive control (see Supplementary Fig. S1). Sixty-nine patient samples and six normal controls were analysed for *IGH* fusions. We identified one patient with *EPOR-IGH*, seven patients with *IGH-DUX4* and one patient with *IGH-ZFP36L2* (see Supplementary Fig. S1). The details of the patients with non-Ph kinase fusions are summarized in Table 2 and Supplementary Table S4.

**Table 2:** Patients with non-Ph kinase fusions.

ID	Age (years)	Gender	WBC (× 10^9^/L)	Fusion gene	Recurrent gene deletions*	MRD	Disease remission	Relapse	BMT	TKI treatment	Outcome
171	17.2	Male	67.1	*EBF1-PDGFRB*	*EBF1* exon 16*, BTG1* exon 2*, IKZF1* exon 2–8	NA	Y	Y	Y	N	Deceased
184	2.7	Male	98.8	*ETV6-ABL1*	No data	NA	Y	N	N	N	Living
299	16.6	Male	310.0	*ZC3HAV1-ABL2*	*CDKN2A/2B, PAX5* exon 2–5*, IKZF1* exon 2–8	NA	Y	Y	N	N	Deceased
362	17.6	Male	49.8	*EPOR-IGH*	*CDKN2A/2B*	NA	Y	Y	Y	N	Deceased
628	15.0	Male	432.0	*CNTRL-ABL1*	*CDKN2A/2B, IKZF1* exon 2–3	NA	N	NA	N	N	Deceased
705	3.9	Male	39.5	*P2RY8-CRLF2*	*CDKN2A/2B*	NA	Y	N	N	N	Living
829	3.7	Male	7.3	*P2RY8-CRLF2*	*IKZF1* exon 4–7	Positive	Y	N	N	N	Living

*WBC* white blood cells, *MRD* minimal residual disease, *BMT* bone marrow transplantation, *TKI* tyrosine kinase inhibitor, *NA* data not available, *N* no, *Y* yes.

*Recurrent gene deletions were identified using the MLPA P335 kit.

### *IKZF1* deletions are enriched in patients with BCR-ABL1, kinase fusions and DUX4 rearrangements

In this cohort, 36 patients harboured *IKZF1* deletions. Of the 14 patients with *BCR-ABL1*, nine (64.3%) had *IKZF1* deletions; of the seven patients with non-Ph kinase fusions, four (57.1%) had *IKZF1* deletions; of the seven patients with *DUX4* rearrangements, five (71.4%) had *IKZF1* deletions. The incidence of *IKZF1* deletions amongst these patients was higher than that amongst the patients without these rearrangements (18/184) (Table 1).

### Survival analysis between patients with non-Ph kinase fusions and other common B-ALL subtypes

**Figure 3:**
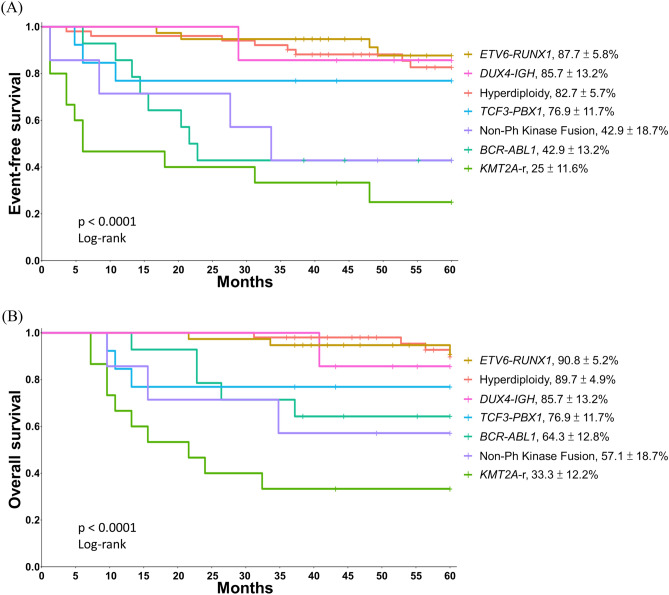
Five-year event-free survival (**A**) and overall survival (**B**) by acute lymphoblastic leukaemia genotype.

Patients with B-ALL were further classified by their genetic markers and frequency: *ETV6-RUNX1* (17.9%), hyperdiploidy (24.1%), *TCF3-PBX1* (6.1%), *KMT2A* (7.0%) and *BCR-ABL1* (6.6%). Favourable factors included *ETV6-RUNX1* (17.9%), hyperdiploidy (24.1%), *TCF3-PBX1* and *DUX4* rearrangements, which were associated with 5-year EFS of 87.7% ± 5.8%, 82.7% ± 5.7%, 76.9% ± 11.7% and 85.7% ± 13.2%, respectively, and 5-year OS of 90.8% ± 5.2%, 89.7% ± 4.9%, 76.9% ± 11.7% and 85.7% ± 13.2%, respectively. Patients with non-Ph kinase fusions, *BCR-ABL1*, and *KMT2A* fusions had worse outcomes: 5-year EFS of 42.9% ± 18.7%, 42.9% ± 13.2% and 25.0% ± 11.6%, respectively, and 5-year OS of 57.1% ± 18.7%, 64.3% ± 12.8% and 33.3% ± 12.2%, respectively. The 5-year OS and EFS of the groups with the different genetic classifications were significantly different (P < 0.0001 and P < 0.0001, respectively; Fig. 3).

## Discussion

In this retrospective study, 67 out of 212 patients without known genetic alterations were included for further analysis. After performing several molecular analyses, including 13 multiplex RT-PCRs, NGS using the kinome kit, and IGH capturing, only five patients with childhood B-ALL were identified with non-Ph kinase fusions, which comprised *EBF1-PDGFRB, ETV6-ABL1, ZC3HAV1-ABL2, EPOR-IGH* and the novel *CNTRL-ABL1*. ALL with non-Ph kinase fusions is associated with inferior 5-year EFS and OS compared with other common subtypes of paediatric ALL, such as *ETV6-RUNX1*, hyperdiploidy and *TCF3-PBX1* and is similar to *BCR-ABL1* fusion.

Amongst B-ALL patients, approximately 10% to 15% of paediatric patients, approximately 25% of adolescents and young adults, and 20% to 30% of adults have Ph-like ALL, based on most reports on Caucasian cohorts^3,4,20–22,24–26,33^. The prevalence rate of Ph-like ALL in Asian cohorts is less well established because of the limited number of studies and small patient populations^9,23,42^. Imamura et al. identified 29 paediatric patients with non-Ph kinase fusions by transcriptome analysis or multiplex RT-PCR analysis in 373 Ph-negative B-ALL patients without recurrent genetic abnormalities in Japan^23^. Another study in China showed the prevalence rate of paediatric Ph-like ALL amongst ALL cases was only 5% by RNA-seq^9^. Rearrangement of *CRLF2* is the most frequent genetic alteration in Ph-like ALL; however, up to 10% of *CRLF2*-rearranged ALL cases have distinctly different gene expression profiles without the activated kinase signature, and thus, are not categorised as Ph-like ALL^43^. In this study, we did not use gene expression to accurately evaluate the prevalence rate of Ph-like ALL. However, using multiplex RT-PCR and kinome and IGH capture sequencing, we identified only five patients with non-Ph kinase fusions and 2 with *P2RY8-CRLF2* amongst the 212 patients with B-ALL. Our methods of analysis failed to detect approximately 10% of Ph-like ALL cases that lack kinase or *CRLF2* rearrangements, according to previous findings^44^. We identified no patients with *IGH-CRLF2*, which might account for approximately 50% patients with *CRLF2* rearrangements. Imamura et al*.* identified ten patients with *P2RY8-CRLF2* and two with *IGH-CRLF2* in 373 paediatric B-ALL without genetic abnormalities^23^. While Yeoh et al*.* used RNA-seq to profile 377 paediatric B-ALL samples and the prevalence rate of Ph-like ALL was only 2%, whereas another 2% had *CRLF2* rearrangements^42^. The lower frequency of paediatric B-ALL with *CRLF2* rearrangements in Asians remains to be determined using more sequencing of larger paediatric ALL cohorts.

Analysis of patient samples using multiplex RT-PCR followed by kinome capture sequencing can identify targetable kinase fusions. However, this approach is labour-intensive and identifies only kinase fusions. The lower incidence of kinase fusion ALL may make this approach inefficient in Taiwan. In addition, after recent efforts in transcriptome sequencing, almost 97% of childhood B-ALL can be classified using RNA-seq, with some prognostic relevance^15,16^. With the progressive decrease in the cost of RNA-seq and the increase in the availability of bioinformatics analysis tools, RNA-seq may represent the best strategy to identify not only kinase fusions but also other novel ALL subtypes such as *ETV6-RUXN1*-like and ALL with *ZNF384* and *MEF2D* fusions^16,21^. Strategies to exclude common recurrent B-ALL, such as that characterised by *ETV6-RUNX1* and hyperdiploidy, could be used to minimise the number of samples submitted for RNA-seq, making it a reasonable, cost-effective approach in Taiwan and other developing countries^38^.

Most retrospective studies reported that Ph-like ALL is associated with worse outcomes than other types of ALL^3,4,20–22,25,26,33,45^. A recent report from Australia and New Zealand demonstrated that despite a risk-adjusted treatment approach, a high rate of relapse persisted amongst children in the ANZCHOG ALL8 study who were retrospectively identified as having Ph-like disease^46^. Imamura et al*.* also demonstrated the inferior prognosis of Ph-like ALL in a Japanese cohort^23^. Previous studies have also indicated that TKIs may be effective for Ph-like ALL^47,48^. Tanasi et al*.* reported, using the largest cohort of patients with ABL-class kinase rearrangements treated with TKI frontline therapy or at relapse, promising minimal residual disease responses and outcomes, reminiscent of those observed in early trials of imatinib combined with chemotherapy in Ph-like ALL^49^. In our study, patients with non-Ph kinase fusions had inferior 5-year EFS and OS, similar to findings previously reported by most studies on Ph-like ALL^3,4,20–22,25,26,33,45^.

Although we identified targetable kinase fusions using this approach, a limitation of this study is that we did not use GEP to identify all Ph-like ALL; identification of Ph-like ALL is based on GEP, although the methods vary^17–19^. Since several possible targetable kinase fusions could be identified in this subtype of B-ALL, GEP-based method is used to screen samples with Ph-like, followed by next-generation sequencing (RNA- or target- seq) or fluorescence in situ hybridization (FISH) to identify fusions^44^. The initial development of low density array (LDA) and/or FISH to identify kinase aimed to shorten the timing for potential patients who might be enrolled into clinical trials^34^. LDA, using 15 genes, was first developed by Harvey et al*.*^50^. The tests were highly sensitive and specific (93% and 89%, respectively) for Ph-like ALL identification. The concordance between this assay and the result of the original 257-gene set analysis was only 87%. Other LDAs with fewer genes were also developed by Heatley et al*.* and Roberts et al*.*^22,46^. Chiaretti et al*.* created a Ph-like ALL predictor by assessing expression of ten genes^51^. Screening for Ph-like ALL by directly searching for specific translocations, similar to this study is also possible. Nonetheless, several cases were detected with Ph-like gene expression signature without detectable driver genetic alterations even with RNA-seq. The actual risk and clinical significance in such cases remain unknown^34^. However, for cost effectiveness, using the RNA-seq might be a better approach than other methods that include multiple steps of screening to identify the targetable kinase fusions^16^. RNA-seq can also classify the subtypes of B-ALL and fusions detections^16^, whereas our sequential approach can identify patients with kinase fusions or *CRLF2* rearrangements although we could not determine whether patients with *CRLF2* rearrangements were Ph-like ALL. This may be resolved using RNA-seq analysis in future prospective studies. Another limitation is the relatively small cohort compared with those of previous studies performed in Caucasian populations. The prevalence rate of B-ALL with kinase fusions observed in this study was considerably lower than that reported in Caucasians, but similar to that reported in other Asian cohorts^9,23,42^. The lower frequency of Ph-like ALL may limit the benefit of just screening Ph-like or kinase fusions in childhood B-ALL in Asian population.

In conclusion, patients with non-Ph kinase fusions have inferior 5-year EFS and OS compared with patients with other common genetic subtypes, in this cohort. The inferior outcomes of the patients with non-Ph kinase fusions supports the use of TKIs in future protocols in Taiwan. Our data suggest that the incidence of Ph-like ALL is low in Taiwan and that other emerging novel subtypes are clinically important. RNA-seq may be a more comprehensive tool for the future genetic analysis of childhood B-ALL in Taiwan.

## Supplementary Information

Supplementary Information.

## Data Availability

The datasets generated during and/or analyzed during the current study are available from the corresponding author on reasonable request.
