# Supplementary material for: Philadelphia chromosome-negative B-cell acute lymphoblastic leukaemia with kinase fusions in Taiwan
Source: Sci Rep. 2021 Mar 11;11:5802. doi: 10.1038/s41598-021-85213-6 (PMC7952704; doi:10.1038/s41598-021-85213-6)
Supplement: Supplementary file 1 — Supplementary Information. [file 41598_2021_85213_MOESM1_ESM.pdf]

**Philadelphia chromosome-negative B-cell acute lymphoblastic leukaemia with kinase fusions in Taiwan**

Yin-Chen Hsu[1*], Chih-Hsiang Yu[1*], Yan-Ming Chen[1], Kathryn G. Roberts[2], Yu-Ling Ni[3], Kai-Hsin Lin[4], Shiann-Tarng Jou[4,5], Meng-Yao Lu[4,5], Shu-Huey Chen[6], Kang-Hsi Wu[7], Hsiu-Hao Chang[4,5], Dong-Tsamn Lin[3,4], Shu-Wha Lin[1,3,8], Ze-Shiang Lin[1], Wei-Tzu Chiu[1], Chia-Ching Chang[1], Bing-Ching Ho[9], Charles G. Mullighan[2], Sung-Liang Yu[1,3,8,9,10], Yung-Li Yang[3,4,11]

[1]Department of Clinical Laboratory Sciences and Medical Biotechnology, College of Medicine, National Taiwan University, Taipei, Taiwan.

[2]Department of Pathology, St. Jude Children's Research Hospital, Memphis, TN, USA.

[3]Department of Laboratory Medicine, National Taiwan University Hospital, Taipei, Taiwan.

[4]Department of Pediatrics, National Taiwan University Hospital, Taipei, Taiwan.

[5]Department of Pediatrics, College of Medicine, National Taiwan University, Taipei, Taiwan.

[6]Department of Pediatrics, Taipei Medical University–Shuang Ho Hospital, Taipei, Taiwan.

[7]Department of Pediatrics, Chung Shan Medical University Hospital and School of Medicine, Chung Shan Medical University, Taichung, Taiwan.

[8]Institute of Medical Device and Imaging, College of Medicine, National Taiwan University, Taipei, Taiwan.

[9]Centers of Genomic and Precision Medicine, National Taiwan University, Taipei, Taiwan.

[10]Graduate Institute of Pathology, College of Medicine, National Taiwan University, Taipei, Taiwan.

[11]Department of Laboratory Medicine, College of Medicine, National Taiwan University, Taipei, Taiwan.

*These authors contributed equally to this article.

**Corresponding authors**:

Yung-Li Yang

Department of Laboratory Medicine, National Taiwan University Hospital, Taipei, Taiwan, 100, No 7. Chung Shan South Road, Email:yangyl92@ntu.edu.tw, Tel: +886-2-23123456-71712, Fax: +886-2-23224263

Sung-Liang Yu

Department of Clinical Laboratory Sciences and Medical Biotechnology, College of Medicine, National Taiwan University, No. 1, Changde Street, Zhongzheng District 10048, Taipei, Taiwan, Email: slyu@ntu.edu.tw, Tel: +886-2-23123456-88697, Fax: +886-223958341

Charles G. Mullighan

Department of Pathology, St. Jude Children's Research Hospital, 262 Danny Thomas Place, Memphis, TN 38105-3678, USA, Email: charles.mullighan@stjude.org, Tel: (901) 595-3387

Fax: (901) 595-5947

Keywords: Ph-like acute lymphoblastic leukaemia, *ABL1* fusion, *CRLF2*, kinase fusion, tyrosine kinase inhibitor

(A)

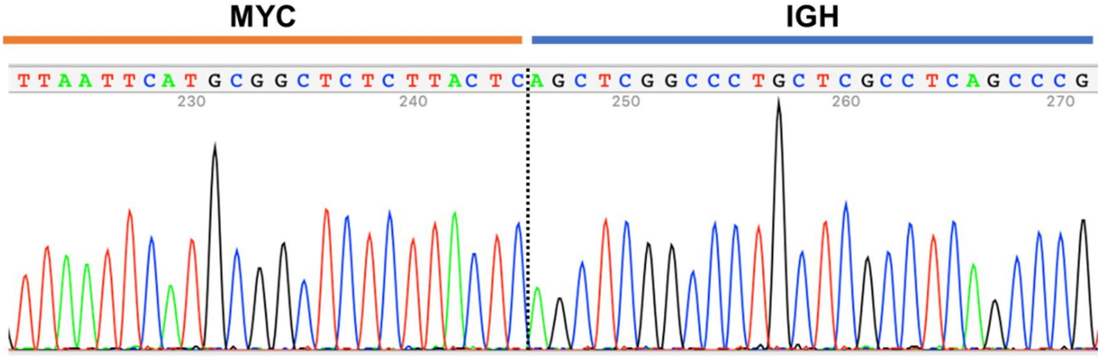
MYC
IGH
TTAATTCATGCGGCTCTCTTACTCAGCTCGGCCCTGCTCGCCTCAGCCCG
230
240
250
260
270

(B)

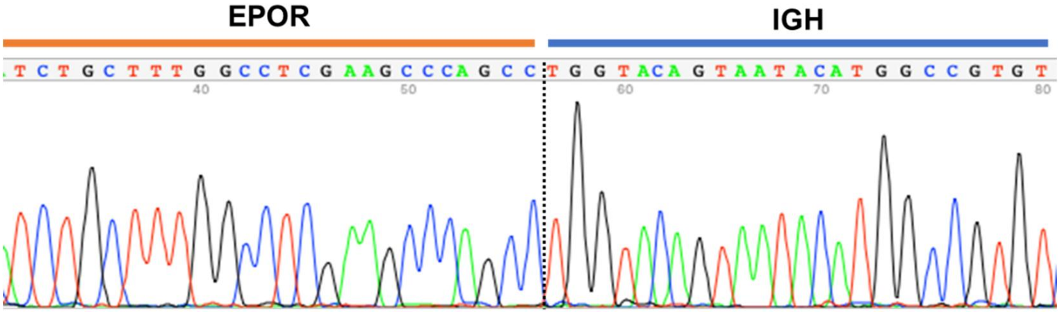
EPOR
IGH
TCTGCTTTGGCCTCGAAGCCCAGCCTGGTACAGTAATACATGGCCGTGT
40
50
60
70
80

(C)

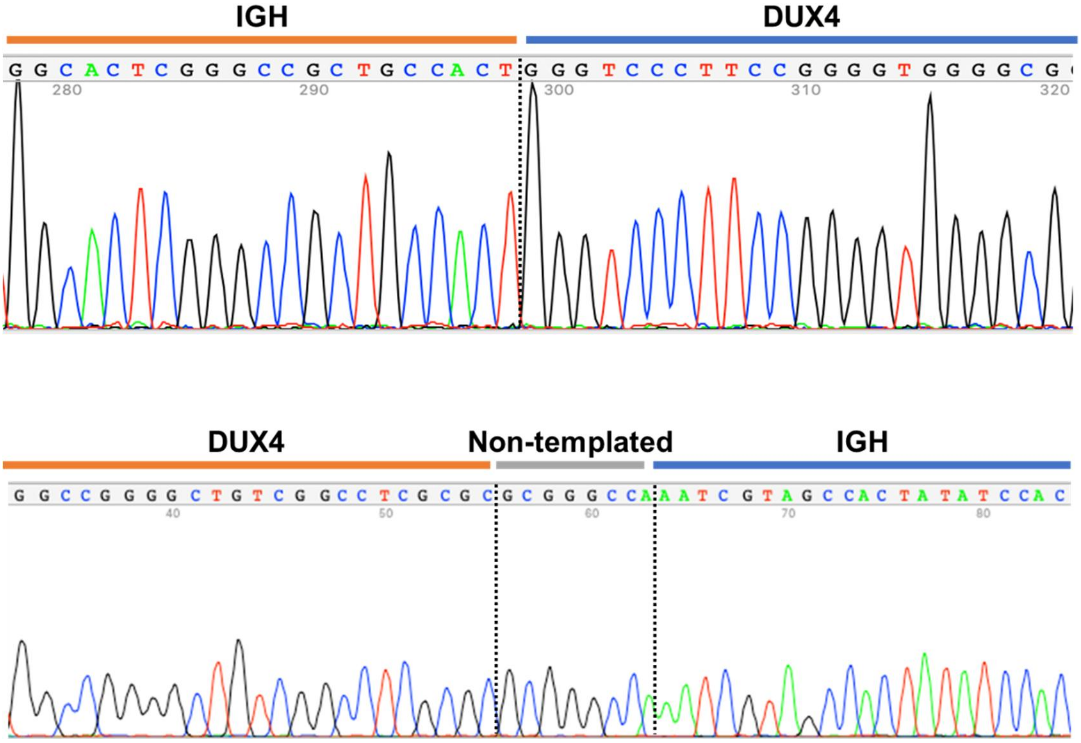
IGH
DUX4
GGCACTCGGGCCGCTGCCACTGGGTCCCTTCCGGGGTGGGGCG
280
290
300
310
320
DUX4
Non-templated
IGH
GGCCGGGGCTGTCGGCCTCGCGCGCGGGCCAAATCGTAGCCACTATATCCAC
40
50
60
70
80

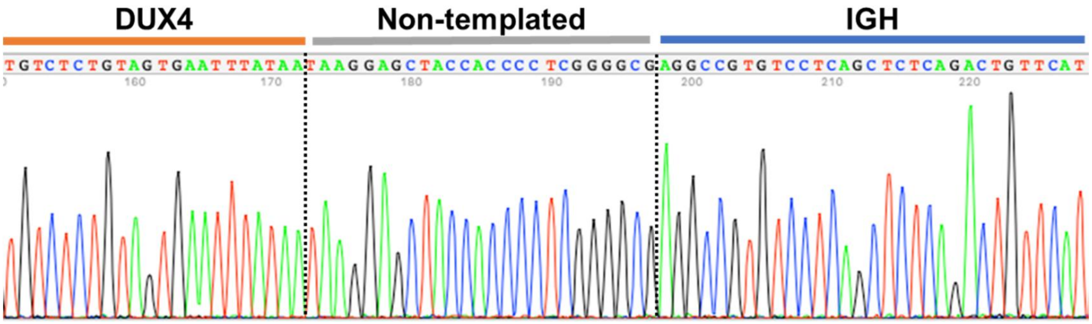


(D)

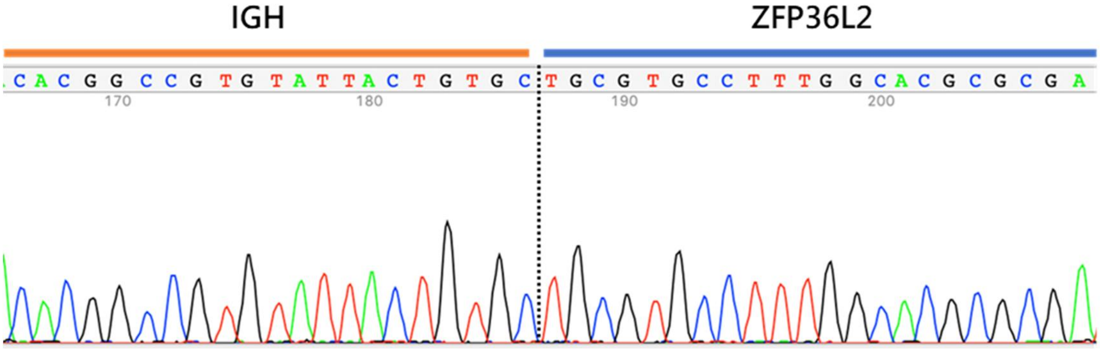


**Supplementary Figure 1.** Examples of (A) *MYC-IGH* (B) *EPOR-IGH* (C) of *DUX4-IGH* and (D) *IGH-ZFP36L2* fusions.

**Supplementary Table 1.** PCR primers for fusion genes.

| **Fusion gene** | **PCR primers (5′ to 3′)** |
|---|---|
| *ETV6-RUNX1* | F: CGTGGATTTCAAACAGTCCA<br>R: CATTGCCAGCCATCACAGTGAC |
| *KMT2A-AFF1* | F: AAAGCAGCCTCCACCACC<br>R: GGTTACAGAACTGACATGCTG |
| *KMT2A-MLLT3* | F: AAAGCAGCCTCCACCACC<br>R: GACCTTGTTGCCTGGTCTGGG |
| *KMT2A-MLLT10* | F: AGCCTCCTCCACGAAAGCCC<br>R: CCCTTTGACCTGAGCTGTGAGC |
| *KMT2A-EPS15* | F: AGTCAGAAACCTACCCCATCAGCAA<br>R: TGGGCCTCCTCAGCACATTTCT |
| *BCR-ABL1* (p190) | F: CGGTTGTCGTGTCCGAGG<br>R: AGATACTCAGCGGCATTG |
| *BCR-ABL1* (p210) | F: CGATGAGGAGCTGGACGCTTT<br>R: CTGCTCTCACTCTCACGCACC |
| *TCF3-PBX1* | F: CAGCCTCATGCACAACCAC<br>R: TAACTCCTCTTTGGCTTCCTC |
| *P2RY8-CRLF2* | F: GCGGCCGCCTTTGCAAGGTTGC<br>R: GTCTAGGAGGCACCCCGAAGTGTGA |
| *TCF3-HLF* (Pt439, Pt784) | F: GCCTGGCAGGAACGTCACAG<br>R: TCAAGTCAGCCACCTCCTGGC |
| *CUX1-NUTM1* (Pt734) | F: CTGACCCCCGAGCAGTACGA<br>R: CCCCAGTGGCCAAGTGAGTC |
| *EPOR-IGH* (Pt362) | F: CCTGGTGGCAGTGTGGACAT<br>R: TGAAGGGCCGATTCACAATCT |
| *CNTRL-ABL1* (Pt628) | F: GTGAGCAGACCCGACTCCAGA<br>R: GTCCGTGCGTTCCATCTCCC |
| *DUX4-IGH* (Pt50) | F: CAGAGCTGGTCAAAGGTGGA<br>R: TTGTAAAGGCCCACAGGCAG |
| *DUX4-IGH* (Pt777) | F: TATGCCATTTTCTCCCTCTATTCTT<br>R: GGTATTAATTGGAATGGTGGTAGCA |
| *DUX4-IGH* (Pt886) | F: GGAGGGCAAAGCCACGC<br>R: ACACTGTGCATGTCTGGTACCTAAG |
| *IGH-ZFP36L2* (Pt787) | F: GAAGCCCGAGAAGCTGAGG<br>R: GGGTTCACCGTCAGTAGCAA |
| *MYC-IGH* (Pt601) | F: CCCTGGGACTCTTGATCAAAG<br>R: TTGTATCAGGCTAAGCCAAGCT |
| *MYC-IGH* (Pt786) | F: CACCGAAGTCCACTTGCCTTT<br>R: GTTTGGGCTGAGCTGGGTTT |
| *MYC-IGH* (Pt797) | F: CGCTATTGACACTTTTCTCAGAGT<br>R: CCGAGCCAGGCTGGTTT |

F, forward; R, reverse.

**Supplementary Table 2.** IGH capture library design.

| Gene name | Chr. | Target-captured regions (hg38) | | Size (bp) |
|---|---|---|---|---|
| | | Start | End | |
| *IGH@* | 14 | 105,566,277 | 106,879,844 | 1,313,567 |
| *BCL2* | 18 | 63,123,346 | 63,320,128 | 196,782 |
| *CEBPA* | 19 | 33,299,934 | 33,302,564 | 2,630 |
| *CEBPB* | 20 | 50,190,583 | 50,192,690 | 2,107 |
| *CEBPD* | 8 | 47,736,909 | 47,739,086 | 2,177 |
| *CEBPE* | 14 | 23,117,304 | 23,119,985 | 2,681 |
| *CEBPG* | 19 | 33,373,330 | 33,382,686 | 9,356 |
| *CRLF2* | X | 1,187,549 | 1,212,815 | 25,266 |
| *EPOR* | 19 | 11,377,205 | 11,384,342 | 7,137 |
| *ID4* | 6 | 19,837,370 | 19,842,200 | 4,830 |
| *IGF2BP1* | 17 | 48,997,412 | 49,056,145 | 58,733 |
| *IGK@* | 2 | 89,851,758 | 90,235,368 | 383,610 |
| *TAL1* | 1 | 47,216,290 | 47,232,373 | 16,083 |
| *TRA@* | 14 | 21,621,904 | 22,552,132 | 930,228 |
| *DUX4* | 4 | 190,173,669 | 190,185,942 | 12,273 |
| *hsa-mir125b-1* | 11 | 122,099,757 | 122,099,844 | 87 |
| *hsa-mir125b-2* | 21 | 16,590,237 | 16,590,325 | 88 |

@, locus; Chr, chromosome.

**Supplementary Table 3.** Comparison of the clinical characteristics of studied and non-studied cohorts

| | Studied | Non-studied | |
|---|---|---|---|
| | n = 212 | n = 142 | P-value# |
| **Gender** | | | 0.38 |
| Male | 121 | 74 | |
| Female | 91 | 68 | |
| **Age (years)** | | | 0.36 |
| <1 | 11 | 5 | |
| 1-9 | 144 | 113 | |
| >10 | 57 | 24 | |
| Median | 5.3 | 5 | |
| Range | 0.01-18.0 | 0.1-17.3 | |
| **WBC (×10$^9$/L)** | | | 0.0002*** |
| <50 | 151 | 115 | |
| 50-100 | 27 | 8 | |
| >100 | 34 | 19 | |
| Median | 18.6 | 9.7 | |
| Range | 0.63-1173.2 | 0.23-1163.4 | |
| **Outcome** | | | 0.14 |
| Living | 137 | 105 | |
| Relapse | 12 | 4 | |
| Deceased | 63 | 33 | |

#: P-value was calculated using Fisher's exact test or the Mann-Whitney test for age and WBC. WBC, white blood cells.

**Supplementary Table 4.** Immunophenotypic information and karyotypes of patients with non-Ph-like fusions.

| ID | CD2 | CD3 | CD5 | CD7 | CD10 | CD13 | CD15 | CD19 | CD20 | CD33 | CD34 | CD56 | CD79a | HLADR | cIg | Kappa | Lambda | Karyotypes |
|---|---|---|---|---|---|---|---|---|---|---|---|---|---|---|---|---|---|---|
| 171 | 2.2 | 1.1 | NA | 0.7 | 96.6 | 0.4 | 0.6 | 95.4 | 19.1 | 13.7 | 68.7 | NA | NA | 95.4 | 90.4 | 0.2 | 0.7 | 46,XY[3] |
| 184 | 4.3 | 1.4 | NA | 1.9 | 97.5 | 52.3 | 1.2 | 96.8 | 96.7 | 2.2 | 46.3 | NA | NA | 95.4 | 2.1 | 5.1 | 2 | 46,XY[2]/46,XY,+9q,11q, acentric(9;20)[17]/46,XY, acentric(9;20)[1] |
| 299 | NA | 0.5 | 0.6 | 0.2 | 99.6 | 13.9 | 2.1 | 97.9 | 47.2 | 26.5 | 32.7 | 0.4 | NA | 93.1 | NA | 0.2 | 2 | 46,XY,t(1,7) |
| 362 | 1.2 | 1.1 | NA | 1 | 98.3 | 2.6 | NA | 98.4 | 37.5 | 30.5 | 95.1 | 6.5 | NA | 89.7 | 0.1 | NA | NA | 46,XY[5]/45,XY,-4p,-7,+9p, -10p,-10q,-14q,+15p[3]/46, XY,+9p,-10p,-10q,-14q[12] |
| 628 | 1.9 | 0.9 | NA | 1.1 | 98.8 | 10.5 | 10.7 | 99.2 | 29.3 | 5.8 | 98.7 | 1 | 98.5 | 99.7 | 13.7 | 3.5 | 2.2 | 47,XY,+9[19]/47,idem,+18, -21[1] |
| 705 | 0.2 | 0.4 | NA | 0.1 | 89.2 | 2.4 | 30.6 | 89.3 | 88.1 | 0.8 | 65.4 | 1.2 | 94.3 | 83.5 | 0.2 | 0.3 | 0 | 46,XY,t(8;9)(q11;p13)[22] |
| 829 | 0.5 | 0.5 | 0.3 | 1.1 | 72.5 | 4.6 | 12.2 | 72.7 | 13.3 | 7 | 76.4 | 0.8 | 78.8 | 59.8 | 1.9 | 0.3 | 0.3 | 46XY[20] |

NA, data not available.
